# A Simple and Rapid Identification Method for *Mycobacterium bovis* BCG with Loop-Mediated Isothermal Amplification

**DOI:** 10.1371/journal.pone.0133759

**Published:** 2015-07-24

**Authors:** Yuji Kouzaki, Takuya Maeda, Hiroaki Sasaki, Shinsuke Tamura, Takaaki Hamamoto, Atsushi Yuki, Akinori Sato, Yasushi Miyahira, Akihiko Kawana

**Affiliations:** 1 Division of Infectious Diseases and Pulmonary Medicine, Department of Internal Medicine, National Defense Medical College, Saitama, Japan; 2 Department of Pediatrics, National Defense Medical College, Saitama, Japan; 3 Department of Laboratory Medicine, National Defense Medical College Hospital, Saitama, Japan; 4 Department of Urology, National Defense Medical College, Saitama, Japan; 5 Department of Global Infectious Diseases and Tropical Medicine, National Defense Medical College, Saitama, Japan; University of Padova, Medical School, ITALY

## Abstract

Bacillus Calmette-Guérin (BCG) is widely used as a live attenuated vaccine against *Mycobacterium tuberculosis* and is an agent for standard prophylaxis against the recurrence of bladder cancer. Unfortunately, it can cause severe infectious diseases, especially in immunocompromised patients, and the ability to immediately distinguish BCG from other *M*. *tuberculosis* complexes is therefore important. In this study, we developed a simple and easy-to-perform identification procedure using loop-mediated amplification (LAMP) to detect deletions within the region of difference, which is deleted specifically in all *M*. *bovis* BCG strains. Reactions were performed at 64°C for 30 min and successful targeted gene amplifications were detected by real-time turbidity using a turbidimeter and visual inspection of color change. The assay had an equivalent detection limit of 1.0 pg of genomic DNA using a turbidimeter whereas it was 10 pg with visual inspection, and it showed specificity against 49 strains of 44 pathogens, including *M*. *tuberculosis* complex. The expected LAMP products were confirmed through identical melting curves in real-time LAMP procedures. We employed the Procedure for Ultra Rapid Extraction (PURE) kit to isolate mycobacterial DNA and found that the highest sensitivity limit with a minimum total cell count of mycobacterium (including DNA purification with PURE) was up to 1 × 10^3^ cells/reaction, based on color changes under natural light with FDA reagents. The detection limit of this procedure when applied to artificial serum, urine, cerebrospinal fluid, and bronchoalveolar lavage fluid samples was also about 1 × 10^3^ cells/reaction. Therefore, this substitute method using conventional culture or clinical specimens followed by LAMP combined with PURE could be a powerful tool to enable the rapid identification of *M*. *bovis* BCG as point-of-care testing. It is suitable for practical use not only in resource-limited situations, but also in any clinical situation involving immunocompromised patients because of its convenience, rapidity, and cost effectiveness.

## Introduction

*Mycobacterium bovis* bacillus Calmette-Guérin (BCG), a live attenuated vaccine derived from a virulent strain of *M*. *bovis* against tuberculosis (TB), has a protective efficacy of 60–80% against severe forms of tuberculosis in children, particularly tuberculous meningitis or miliary tuberculosis [1,2]. BCG is also used as immunotherapy for bladder cancer, where it improves the risk of recurrence and disease progression compared with chemotherapy [3]. It is considered a safe, low-cost, and easily produced vaccine with a low overall incidence of complications (estimated at ≤1%) [4]. However, serious local complications of BCG vaccinations, such as adenitis, abscesses, and fistulae, have been reported even in healthy individuals. Additionally, other infectious systemic complications have also been reported, such as osteitis [5], cystitis, genitourinary infection, hepatitis, and pneumonitis [6]. The predisposing factors for these severe complications include underlying severe combined immunodeficiency [7] and AIDS [8]. The prognosis of disseminated BCG disease is poor, even when treatment is aggressive, due to associated immunodeficiency, delays in diagnosis, and initial treatment with pyrazinamide to which BCG is uniformly intrinsic resistant even though it is a first-line anti-tuberculosis drug [9]. Therefore, it is important to obtain precise and rapid identification of *M*. *bovis* BCG to distinguish it from other *M*. *tuberculosis* complex (*Mtb* complex) subspecies including *M*. *tuberculosis*, *M*. *africanum*, *M*. *bovis*, and *M*. *microti* [10] for both the treatment of patients and the collection of significant epidemiological information on the safety of the vaccination.

Detection procedures based on classical conventional culture methods and biochemical features, such as the nitratase activity test, oxygen preference test, the niacin test, and tests for susceptibility to thiophen-2-carboxylic acid hydrazide, pyrazinamide, and pyrazinamidase activity are considered gold standard tools for obtaining definitive diagnosis of TB [11]. However, these methods cannot distinguish *M*. *bovis* BCG from other species of the *Mtb* complex and are time-consuming. An automatic cultivation and detection system called BACTEC Mycobacterial Growth Indicator Tube (MGIT) 960 (Nippon Becton Dickinson, Tokyo, Japan), which is widely used around the world, improves the time required for the detection of mycobacteria [12]. However, similarly to conventional culture methods, this well-known method cannot differentiate the species of the *Mtb* complex. More sophisticated methods for the identification of BCG include conventional culture, followed by phage typing [13], high-performance liquid chromatography (HPLC) [14], IS*1081* fingerprinting [15], and PCR-restriction enzyme analysis of the major polymorphic tandem repeats [16]; however, these methods are not easy to use.

A comparative genomic study to identify the genetic differences between *M*. *tuberculosis* H37Rv and *M*. *bovis* BCG revealed 16 deleted regions in *M*. *bovis* BCG strains [17]. Region of difference 1 (RD1) is missing from all *M*. *bovis* BCG strains, thus it has become an accurate and reliable marker for molecular diagnosis. Several methods based on the polymerase chain reaction (PCR) for identification of *Mtb* complex have since been reported [18,19,20]. Consequently, we have sophisticated and powerful tools available for clinical and epidemiological work on *Mtb* complex. However, these methods require experience, expertise and expensive laboratory equipment, all of which create obstacles for their use in settings with limited clinical resources. There remains an urgent need to facilitate and simplify the identification procedures, so they can be completed without any specialized knowledge or expensive equipment for the specific diagnosis of *M*. *bovis* BCG.

A novel loop-mediated isothermal amplification (LAMP) method, which does not require expensive devices or instruments, was first introduced in 2000 [21]. This method employs a set of four specially designed primers that recognize six distinct sequences on the target DNA, and relies on an auto cycling procedure performed with *bst* DNA polymerase. Therefore, it could offer both high sensitivity and specificity, and be extremely convenient to use. The procedure has already been evaluated for the diagnosis of various infectious diseases: TB [22,23], extrapulmonary tuberculosis [24], malaria [25,26], cutaneous leishmaniasis [27], and pneumocystis pneumonia [28]. The LAMP method could become a widely-used diagnostic technique that requires only limited equipment and manpower because it is suitable for use not only in resource-limited situations but also in well-equipped health facilities.

In this study, we developed a simple and rapid identification method for *M*. *bovis* BCG using the LAMP procedure. We also combined it with a porous material in the form of the commercially available Procedure for Ultra Rapid Extraction (PURE) kit (Eiken Chemical, Tokyo, Japan) to rapidly isolate DNA in a simple way as point-of-care testing. This approach is anticipated to lead to a LAMP-based definitive diagnosis of *M*. *bovis* BCG infections.

## Materials and Methods

### Preparation of genomic DNA for molecular analysis

Genomic DNA of *M*. *bovis* BCG was prepared from a clinical isolate obtained from a patient with vesical tuberculosis after BCG intravesical instillation therapy for prophylaxis against bladder cancer and was identified at the Research Institute of Tuberculosis, Japan Anti-tuberculosis Association. Mycobacterial genomic DNA was extracted from bacteria grown on Ogawa medium (Kyokuto Pharmaceutical, Tokyo, Japan) with an Isoplant kit (Nippon gene, Toyama, Japan) using the following procedure. Briefly, isolated colonies from Ogawa medium were suspended with 300 μL of provided extraction buffer and incubated overnight at 37°C before further incubation for 40 min at 52°C with lysis buffer (150 μL). Cell lysates were extracted with sodium acetate and ethanol. The purified genomic DNA was suspended in 100 μL of 1× TE and the final concentration was adjusted from 1.0 fg/μL to 1.0 ng/μL as templates for LAMP.

### Design of LAMP primers

**Fig 1:**
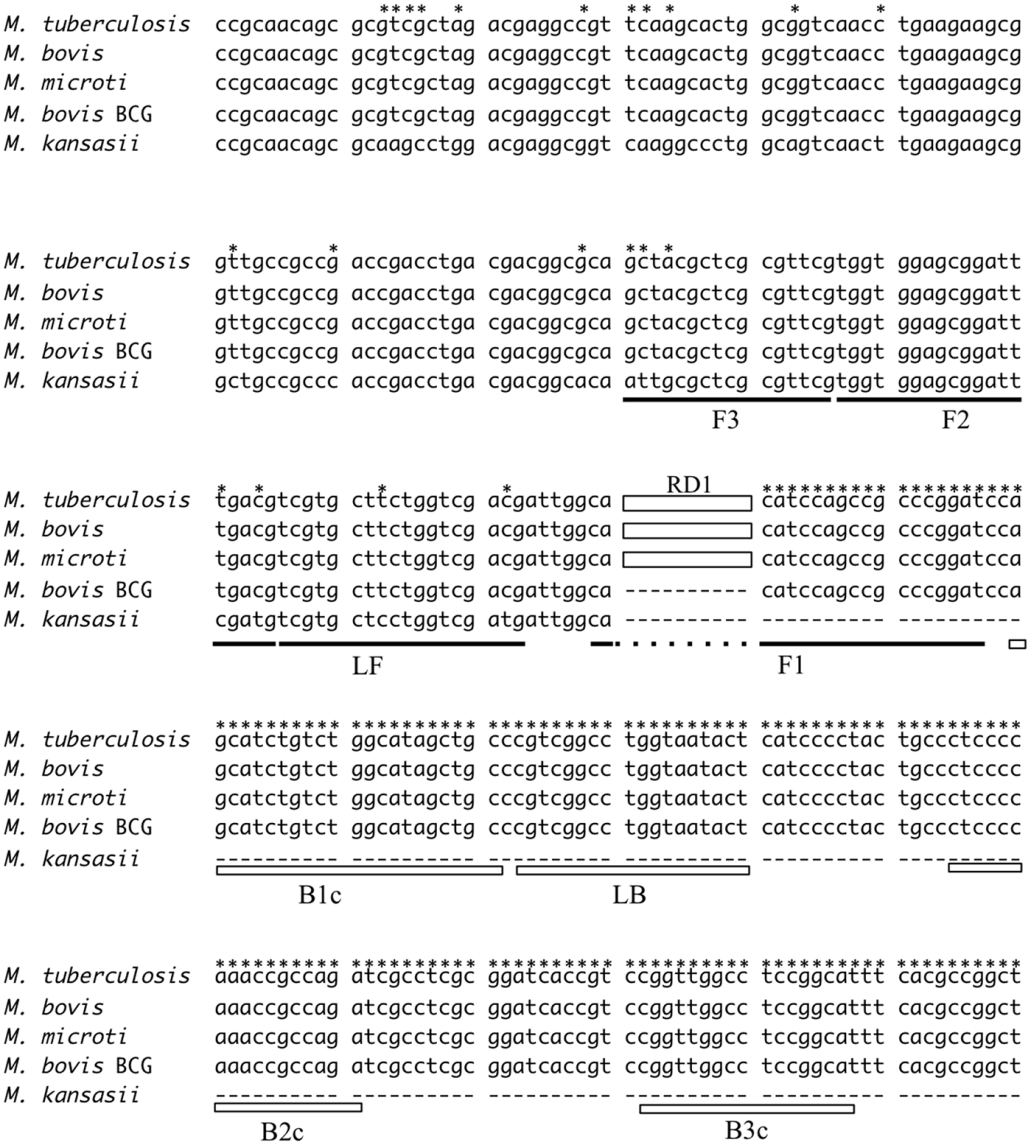
Alignment of the sequences including RD1, which encodes a 9.5-kb fragment and is deleted in *M*. *bovis* BCG. The constructed sets of LAMP primers are shown as lines and boxes. The segment of the RD1 sequence, depicted as solid-white boxes and named RD1, is inserted within *M*. *tuberculosis*, *M*. *bovis*, and *M*. *africanum* sequences. Asterisks show the specific conserved sequences within the *M*. *tuberculosis* complex.

The oligonucleotide LAMP primers for specific detection of the *M*. *bovis* BCG genes were designed based on the RD1 sequence (GenBank accession no. AP010918.1). Genbank sequences initially considered were tested *in silico* through BLAST searches and alignment analysis [29]. Several candidate primer sets were suggested *via* online LAMP primer design software (PrimerExplorer 4, http://primerexplorer.jp/index.html; Eiken Chemical, Tokyo, Japan), and further refinement of primer design was developed manually based on the criteria described in “A Guide to LAMP primer designing” (http://primerexplorer.jp/e/v4_manual/index.html). Each selected primer sequence is given in Table 1 and their positions are shown in Fig 1.

**Table 1:** Nucleotide sequences of designed LAMP primers. F3 and B3; Outer primers, FIP and BIP; Inner primers, FL and BL; loop primers.

Primer type	Sequences (5′ - 3′)	Length
F3	GCTACGCTCGCGTTCG	16
B3	ATGCCGGAGGCCAACC	16
FIP*	ATCCGGGCGGCTGGATGTGTGGTGGAGCGGATTTGACG	38
BIP**	AGCATCTGTCTGGCATAGCTGCATCTGGCGGTTTGGGGAG	40
LF	CGTCGACCAGAAGCACGA	18
LB	CCGTCGGCCTGGTAATACT	19

*The FIP primer consisted of F2 and the complementary strand of F1 (F1c).

**The BIP primer consisted of B2 and the complementary strand of B1 (B1c).

### LAMP reactions

The reactions were performed using a Loopamp DNA Amplification Reagent Kit (Eiken Chemical), detection was performed by real-time measurement of turbidity using a Loopamp EXIA (Eiken Chemical), and visual observation with the naked eye. DNA samples (1 μL) were subjected to amplification in reaction mixtures, each of which contained 40 pmol of the FIP and BIP primers, 20 pmol of the each loop primer, 5 pmol of F3 and B3 primers, 1 μL of provided *Bst* DNA polymerase, and reaction buffer (20 mM of Tris-HCl, 10 mM of KCl, 8 mM of MgSO_4_, 10 mM of (NH_4_)_2_SO_4_, 0.1% of Tween 20, 0.8 M of betaine, and 1.4 mM of each dNTP), with or without 1 μL of fluorescent detection reagent (FDR) (Eiken Chemical). Distilled water was added to bring the final volume of 25 μL. Successful targeted gene amplifications without FDR were detected by real-time measurement of turbidity with a Loopamp EXIA, and those with FDR were detected by the naked eye from color changes under natural light. Reactions were carried out at 61°C, 62°C, 63°C, and 64°C in triplicate to find the optimum temperature for LAMP amplification.

### Sensitivity and specificity of the LAMP assay

The sensitivity of the LAMP assay was determined using ten-fold serially diluted genomic DNA of *M*. *bovis* BCG ranging from 1.0 ng/μL to 1.0 fg/μL and stored at −20°C in triplicate until required. Additionally, to evaluate the specificity of the test, we also tested 49 kinds of pathogens isolated by the National BioResource Project (http://www.nbrp.jp/) (Table 2).

**Table 2:** Bacterial and fungal pathogens used to determine specificity.

Bacteria (GTC numbers; Name)			Fungus (IFN numbers; Name)		
*CL* *	*Mycobacterium*	*tuberculosis*	*4924*	*Aspergillus*	*fumigatus*
*12853*	*M*.	*microti*	*41398*	*A*.	*niger*
*00614*	*M*.	*kansasii*	*5366*	*A*.	*flavus*
*00603*	*M*.	*avium*	*5505*	*Cryptococcus*	*neoformans*
*00612*	*M*.	*gordonae*	*61880*	*C*.	*gattii*
*00613*	*M*.	*intracellulare*	*4949*	*Candida*	*albicans*
*00616*	*M*.	*marinum*	*5489*	*C*.	*glabrata*
*15340*	*M*.	*chelonae*	*5462*	*C*.	*krusei*
*15378*	*M*.	*fortuitum*	*5464*	*C*.	*parapsilosis*
*15673*	*M*.	*scrofulaceum*	*5446*	*C*.	*tropicalis*
*00980*	*Streptococcus*	*pneumoniae*	*40507*	*Mucor*	*circinelloides*
*01524*	*Staphylococcus*	*aureus*	*40515*	*Rhizopus*	*oryzae*
*15087*	*Klebsiella*	*pneumoniae*	*0138*	*Nocardia*	*farcinica*
*03814*	*Haemophilus*	*influenza*	*55089*	*Spirotrichum*	*purpureum*
*03941*	*H*.	*influenza*	*49269*	*Basidiobolus*	*meristosporus*
*10424*	*H*.	*influenza*	*5279*	*Trichophyton*	*violaceum*
*10426*	*H*.	*influenza*	*40746*	*Epidermophyton*	*floccosum*
*14643*	*Acinetobactor*	*baumanii*	*41980*	*Microsporum*	*ferrugineum*
*00745*	*Legionella*	*pneumophila*	*4805*	*Cladosporium*	*carrionii*
*3p0017*	*Burkholderia*	*pseudomallei*	*40081*	*Malassezia*	*furfur*
*3p0025*	*B*.	*pseudomallei*	*46458*	*Madurella*	*mycetomi*
*3p0056*	*B*.	*pseudomallei*	*4856*	*Fonsecaea*	*pedrosoi*
*03411*	*Escherichia*	*coli*	*40732*	*Trichophyton*	*rubrum*
*13866*	*Pseudomonas*	*aeruginosa*	*CL* *	*Pneumocystis*	*jirovecii*
*14757*	*Proteus*	*mirabilis*			

*CL; clinically isolated strain.

### Analysis of LAMP products

To evaluate the presence of any non-specific products, we performed a melting curve analysis on the real-time LAMP products in a 96-well plate on a Roche LightCycler LC480 System (Roche Diagnostics GmbH, Mannheim, Germany), according to a previously reported protocol [28,30]. The reaction mixtures contained 20 mU Tth pyrophosphatase per mL (New England Biolabs, MA, USA) and 0.25 μg YO-PRO-1 per mL (Invitrogen, Carlsbad, CA, USA). Reactions were performed at 63°C for 60 min and then heated at 80°C for 2 min to terminate them. The temperature transition rate was 0.2°C per s, increasing from 60–98°C, and the cooling temperature for melting curve analysis was 30°C for 1 min. The instrument’s software automatically calculated the melting peaks.

### Analysis of the detection limit for artificial clinical specimens containing mycobacterial suspensions

We applied the PURE kit, which is designed for the rapid DNA purification of mycobacterium from sputum without requiring additional laboratory equipment, to isolate mycobacterial DNA from several clinical specimens [23]. To evaluate the effectiveness of our procedure in clinical practice for point-of-care testing, we constructed 10 sets of artificial clinical samples containing serially diluted solutions of *M*. *bovis* BCG (GTC no. 00499) cultured with Ogawa medium (Kyokuto Pharmaceutical, Tokyo, Japan) and adjusted to McFarland standards from 1.0 × 10^1^–1.0 × 10^7^ cells/mL. In this study, we used serum, urine, and cerebrospinal fluid (CSF) provided as aseptic human specimens (Biopredic International, Saint-Gregoire, France) as clinical vehicles. Additionally, 10 bronchoalveolar lavage fluid (BALF) samples were collected from non-mycobacterial patients, stored at -80°C until the day of processing, evenly mixed, and used as a clinical vehicle. Blood contamination in clinical specimens can make it difficult to verify reactivity based on turbidity and can also inhibit the amplification step of the LAMP reaction [31]; therefore, to evaluate the effect of blood contamination on the sensitivity of the LAMP reaction, we also prepared several blood contaminated solutions diluted stepwise by 20% with saline from 0% to 100%. Finally, 100 μL of artificially created sample was further mixed with 900 μL of DNA extraction solution provided with the PURE kit and was heated on a heating block at 100°C for 10 min. DNA was purified through a porous material provided with the PURE kit following the manufacturer’s instructions. After purification, 1 μL of each extracted DNA sample was subjected to the LAMP procedure with FDR protocol and was followed by identification of the product by naked eye in triplicate (Table 3). This study was approved by the National Defense Medical College Research Ethics Committee (reference 2310).

**Table 3:** Reactive pattern of LAMP with PURE for artificially pulsed *M*. *bovis* BCG.

PURE/LAMP samples	Amount of pulsed control mycobacterium (cells/reaction)							
	NC*	10^0^	10^1^	10^2^	10^3^	10^4^	10^5^	10^6^
NS** 100 μL	0/3	0/3	0/3	0/3	3/3	3/3	3/3	3/3
NS 80 μL/Blood 20 μL	0/3	0/3	0/3	0/3	3/3	3/3	3/3	3/3
NS 60 μL/Blood 40 μL	0/3	0/3	0/3	0/3	2/3	3/3	3/3	3/3
NS 40 μL/Blood 60 μL	0/3	0/3	0/3	0/3	0/3	3/3	3/3	3/3
NS 20 μL/Blood 80 μL	0/3	0/3	0/3	0/3	0/3	0/3	3/3	3/3
Blood 100 μL	0/3	0/3	0/3	0/3	0/3	0/3	0/3	0/3
Serum 100 μL	0/3	0/3	0/3	0/3	2/3	3/3	3/3	3/3
Urine 100 μL	0/3	0/3	0/3	0/3	3/3	3/3	3/3	3/3
CSF*** 100 μL	0/3	0/3	0/3	0/3	3/3	3/3	3/3	3/3
BALF 100 μL	0/3	0/3	0/3	0/3	3/3	3/3	3/3	3/3

*NC; negative control,

**NS; normal saline,

***CSF; cerebrospinal fluid.

## Results

### Optimization of specific LAMP reactions

Amplification was performed at a range of temperatures (61–64°C) with or without FDA reagents to determine the optimal incubation temperature for the LAMP reactions. The optimal temperature was found to be 64°C for both procedures and LAMP products were detected within 30 min.

### Sensitivity of LAMP

**Fig 2:**
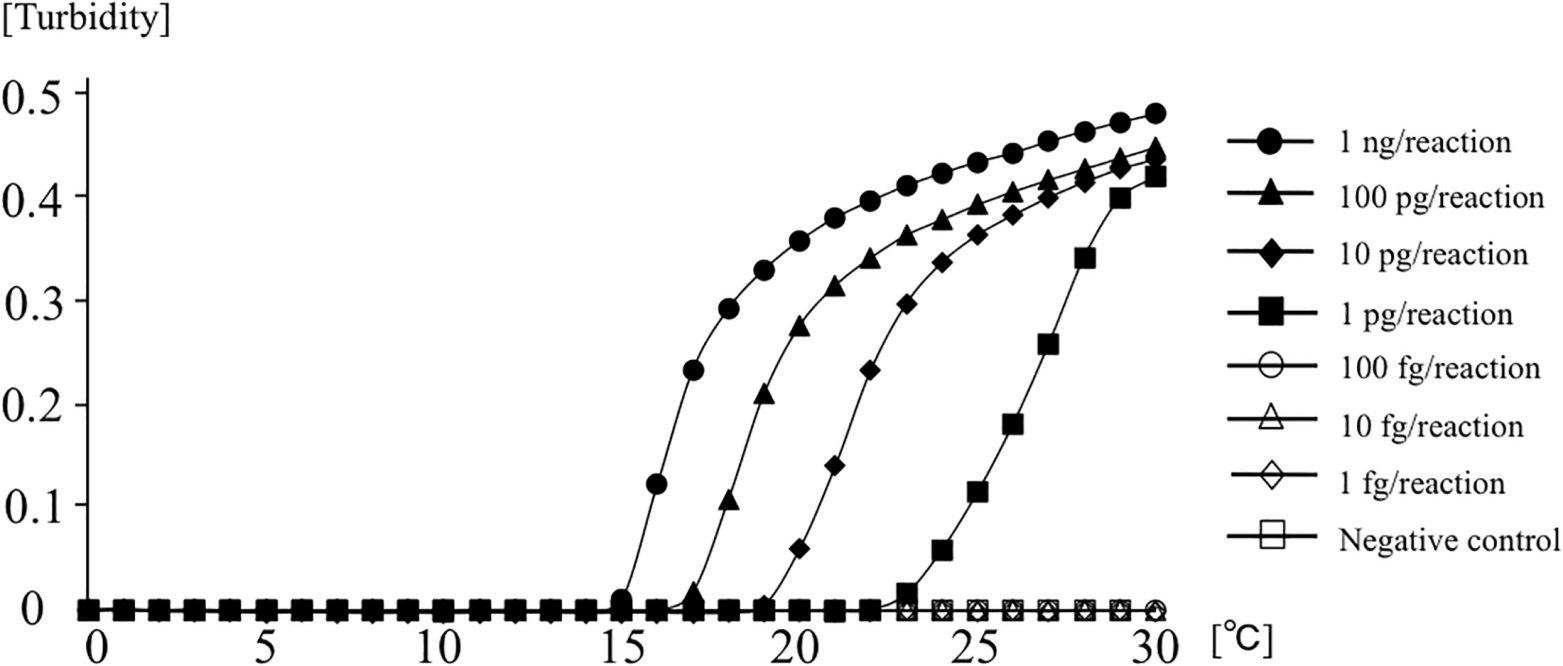
The sensitivity of LAMP for the detection of *M*. *bovis* BCG with Loopamp EXIA.

**Fig 3:**
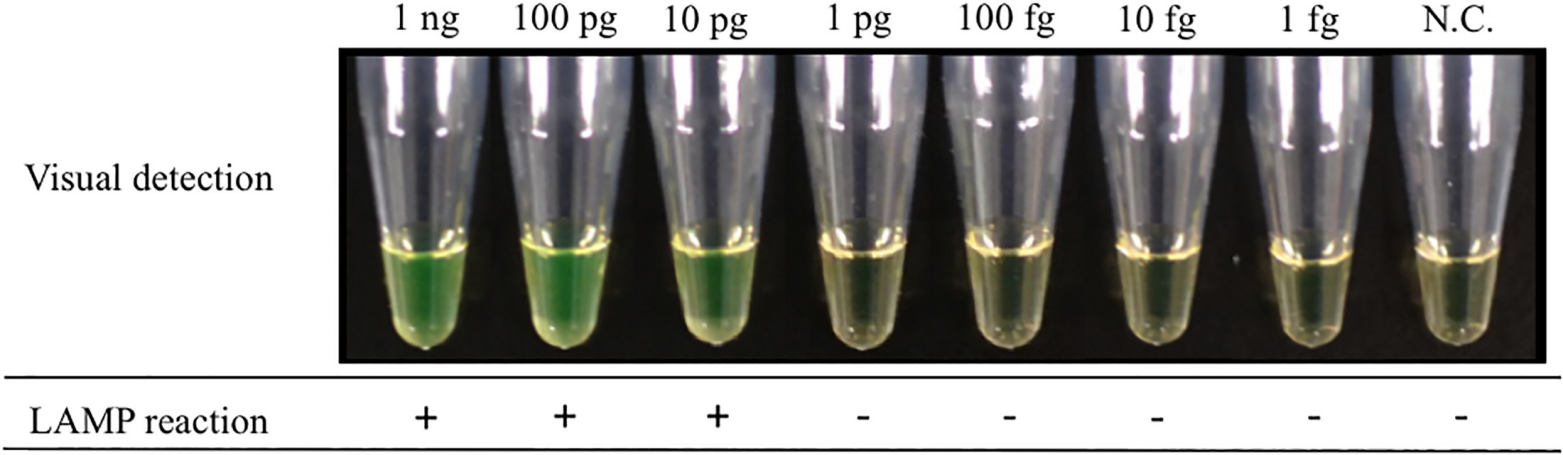
The sensitivity of the LAMP for the detection of *M*. *bovis* BCG in the visual detection of LAMP products under natural light.

A 10-fold serial dilution of genomic DNA (1 ng/reaction to 1 fg/reaction) from *M*. *bovis* BCG was amplified to determine the lower limit of detection in reactions at 64°C. Fig 2 shows the results of detection of real-time turbidity with a Loopamp EXIA; amplification of the target DNA is indicated by the rising curve. The results of fluorescence detection based on color changes under natural light with FDA reagents are shown in Fig 3. A color shift from pale brown to bright fluorescent green represented successful amplification of the target gene. The minimum amount of on real-time turbidity with a Loopamp EXIA DNA was 1 pg/reaction, whereas the visual color change was one log less sensitive to 10 pg/reaction in the presence of FDR (Figs 2 and 3). Detection was achieved within 30 min in both procedures.

### Specificity of LAMP

Positive results were obtained using only purified DNA from *M*. *bovis* BCG, whereas other purified DNA samples from all other 49 strains of 44 pathogenic species, including another species in the *Mtb* complex (*M*. *tuberculosis* and *M*. *microti*) were not amplified. All samples were tested in triplicate.

### Analysis of LAMP products

**Fig 4:**
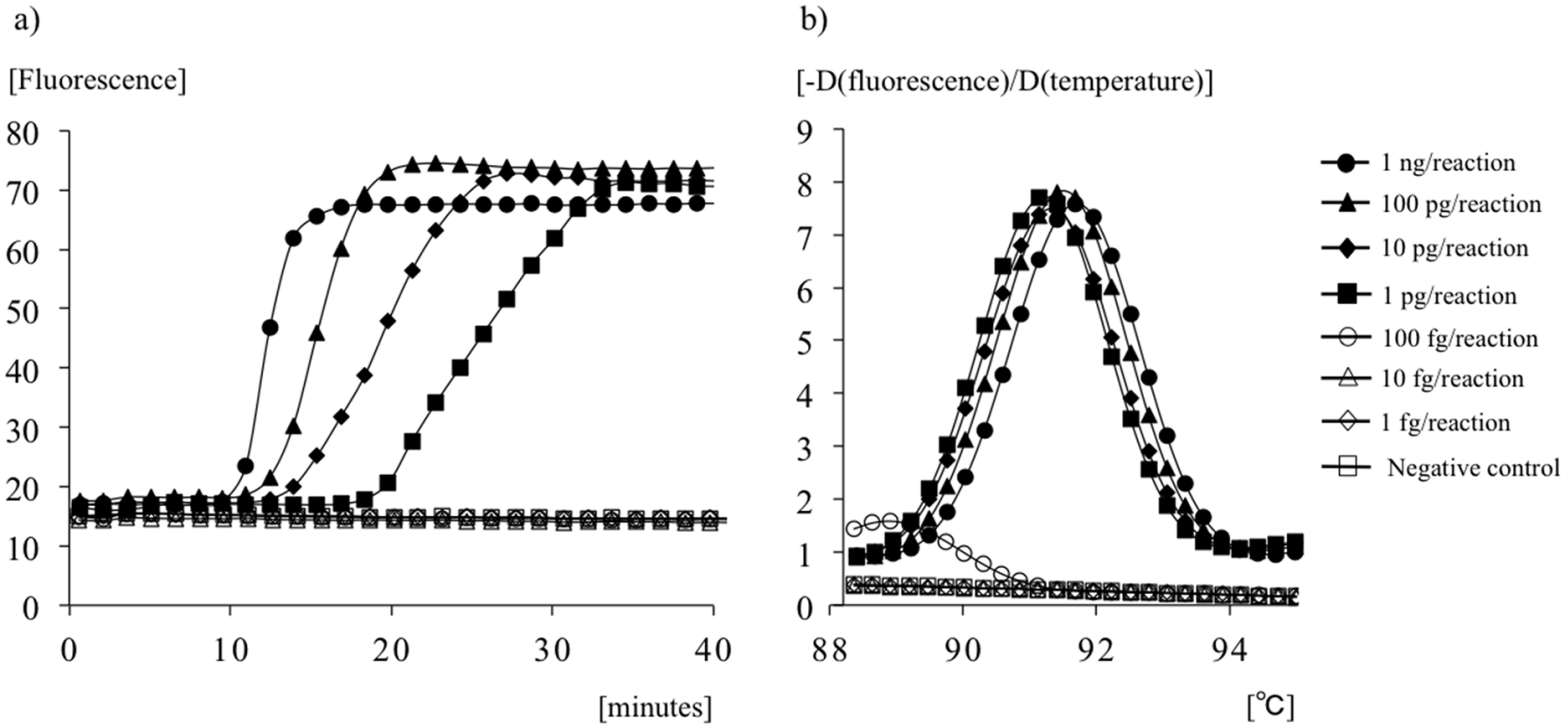
Analysis of the LAMP assay with a LightCycler. (a) Amplification analysis; the rising curves of fluorescence indicate DNA amplification. (b) Melting-curve analysis; the melting temperature of each sample is shown as a peak.

Real-time LAMP reactions containing Tth pyrophosphatase and YO-PRO-1 could detect the genomic DNA of *M*. *bovis* BCG at 1 pg/reaction within 30 min (Fig 4a). All melting curve peaks (1 ng to 1 pg/reaction) obtained under this procedure were consistent (Fig 4b). Although this protocol did not produce non-specific reactions within 30 min, reactions of less than 100 fg/reaction for more than 30 min yielded melting curve peaks that differed from those seen in specific reactions.

### Performance of the LAMP procedure with PURE

Table 3 shows the potential capacity of our LAMP procedure combined with PURE to purify the DNA from mycobacterial suspension up to 1.0 × 10^4^ cells/mL, achieving positive amplification. Because 100 μL of each specimen was used to purify its DNA in our method, we were able to detect *M*. *bovis* BCG from mycobacterial suspensions equivalent to 1.0 × 10^3^ cells/reaction based on color changes under natural light with FDA reagents. The PURE-LAMP methods could also directly detect approximately 1.0 × 10^3^ cells/reaction, even in clinical vehicles comprising serum, urine, CSF and BALF. Furthermore, inclusion of up to 20% blood in the clinical specimens could be reliably removed using this rapid purification procedure.

## Discussion

In the present study, a LAMP assay was successfully developed with a set of original six primers targeted to the RD1 deletions of *M*. *bovis* BCG. Previous reports showed that genome sequences containing the RD1 gene are completely conserved in *Mtb* complex subspecies, including in *M*. *tuberculosis*, *M*. *africanum*, *M*. *bovis*, and *M*. *microti*. Therefore, the absence of the target RD1 gene could be used for the specific identification of *M*. *bovis* BCG. In the specificity evaluation test conducted in this study, no false-positive results were found for any other bacterial or fungal samples, including all non-tuberculous mycobacteria. In particular, amplification on *M*. *tuberculosis* and *M*. *microti* were completely negative. Analysis of the melting curve peaks following real-time LAMP reactions could support the specificity of the amplified product with LAMP and ensure correct identification of *M*. *bovis* BCG. In the sensitivity evaluation test, we found that the detection limit of this LAMP procedure with purified DNA was 1.0 pg/reaction within 30 min with detection of real-time turbidity with an EXIA turbidimeter and real-time fluorescence on a LightCycler LC480 System. Gene amplification with LAMP also allowed simple detection by end-point visual inspection of fluorescence without the use of a turbidimeter or thermal cycler and could therefore eliminate the need for extensive training or a stable electrical supply. In this study, confirmations of LAMP products based on this visual color change with FDR were only one log less sensitive, and this method could detect up to 10 pg/reaction of BCG genome within 30 min. In a previous report, the detection limit for *M*. *bovis* was 10 pg/reaction by conventional PCR and 1 pg/reaction by LAMP targeting the *rim*-encoding 16S rRNA-processing protein [32]. Our results are in accordance with these methods and could provide adequate performance for clinical specimens.

The LAMP procedure shares a characteristic with other nucleic acid amplification tests (NAATs) in that it requires DNA template purification from clinical samples. However, compared with other NAATs, such as PCR, the LAMP assay is more resistant to contamination with reaction-inhibitory substances often found in clinical samples. Eiken Chemical Company and Foundation for Innovative New Diagnostics successfully developed the TB-LAMP kit, which was combined with the PURE kit for the purification of clinical samples [33]. The commercially available PURE device consists of interlocking plastic components and provides a closed system for rapid DNA purification of mycobacterium from clinical samples without the need for any other laboratory equipment (e.g., micropipettes or centrifuges). The device has also contributed to minimization of contamination. LAMP with the PURE test consists of three steps: sample preparation, amplification with LAMP, and visual detection of fluorescence under natural light. These three steps can be completed for diagnosis of TB in less than 1 h [23,34]. Therefore, the PURE method has recently been applied as a sophisticated, simple, and rapid purification tool for various clinical samples: blood [25,26], debris from cutaneous ulcers or purulent aspirate [24,31,35], and bronchoalveolar lavage fluid [36] including pathogens such as mycobacteria, protozoa, and fungi. These reports showed that contamination from blood, purulent discharge, and respiratory tract secretions in the LAMP assay were negligible even for making a diagnosis with the naked eye.

In the present study, the minimum total cell count of mycobacterium including DNA purification with PURE that was sufficient for detection based on color changes under natural light with FDA reagents was equivalent to 1 × 10^3^ cells/reaction. Therefore, the conventional culture followed by LAMP combined with PURE could be sufficient to allow for the quick estimation of isolated mycobacterium from clinical samples. Additionally, the PURE procedure could directly extract DNA from not only culture medium, but also from several clinical samples while eliminating inhibitory substances to make a simple diagnosis of BCG infections, except in the case of bloodstream infections. Thus, our procedure could serve as a powerful tool for identification of *M*. *bovis* BCG not only in resource-limited situations such as small-scale hospitals or in field settings, but also in well-equipped health facilities in developed countries.

In conclusion, we produced a LAMP procedure for identifying *M*. *bovis* BCG, which allows complete testing from sample preparation to confirmation of the reactions using minimum resources and results are achievable within a short period and with high detection sensitivity. The next step for refining this procedure will be to conduct a clinical evaluation to ensure sufficient and consistent sensitivity and specificity.
